# The Effect of Chromosome 9p21 Variants on Cardiovascular Disease May Be Modified by Dietary Intake: Evidence from a Case/Control and a Prospective Study

**DOI:** 10.1371/journal.pmed.1001106

**Published:** 2011-10-11

**Authors:** Ron Do, Changchun Xie, Xiaohe Zhang, Satu Männistö, Kennet Harald, Shofiqul Islam, Swneke D. Bailey, Sumathy Rangarajan, Matthew J. McQueen, Rafael Diaz, Liu Lisheng, Xingyu Wang, Kaisa Silander, Leena Peltonen, Salim Yusuf, Veikko Salomaa, James C. Engert, Sonia S. Anand, on behalf of the INTERHEART investigators

**Affiliations:** 1 Department of Human Genetics, McGill University, Montréal, Quebec, Canada; 2 Population Health Research Institute, Hamilton Health Sciences, and McMaster University, Hamilton, Ontario, Canada; 3 Departments of Medicine and Clinical Epidemiology and Biostatistics, McMaster University, Hamilton, Ontario, Canada; 4 Department of Chronic Disease Prevention, National Institute for Health and Welfare, Helsinki, Finland; 5 Estudios Cardiologicos Latinoamerica, Rosario, Argentina; 6 Cardiovascular Institute and Fu Wai Hospital, Chinese Hypertension League Institute, Beijing, China; 7 Laboratory of Human Genetics, Beijing Hypertension League Institute, Beijing, China; 8 Human Genomics Unit, Institute for Molecular Medicine Finland (FIMM), University of Helsinki, Helsinki, Finland; 9 Department of Medicine, McGill University, Montréal, Quebec, Canada; 10 The Research Institute of the McGill University Health Centre, Montréal, Quebec, Canada; Kings College London, United Kingdom

## Abstract

**Background:**

One of the most robust genetic associations for cardiovascular disease (CVD) is the Chromosome 9p21 region. However, the interaction of this locus with environmental factors has not been extensively explored. We investigated the association of 9p21 with myocardial infarction (MI) in individuals of different ethnicities, and tested for an interaction with environmental factors.

**Methods and Findings:**

We genotyped four 9p21 SNPs in 8,114 individuals from the global INTERHEART study. All four variants were associated with MI, with odds ratios (ORs) of 1.18 to 1.20 (1.85×10^−8^≤*p*≤5.21×10^−7^). A significant interaction (*p* = 4.0×10^−4^) was observed between rs2383206 and a factor-analysis-derived “prudent” diet pattern score, for which a major component was raw vegetables. An effect of 9p21 on MI was observed in the group with a low prudent diet score (OR = 1.32, *p* = 6.82×10^−7^), but the effect was diminished in a step-wise fashion in the medium (OR = 1.17, *p* = 4.9×10^−3^) and high prudent diet scoring groups (OR = 1.02, *p* = 0.68) (*p* = 0.014 for difference). We also analyzed data from 19,129 individuals (including 1,014 incident cases of CVD) from the prospective FINRISK study, which used a closely related dietary variable. In this analysis, the 9p21 risk allele demonstrated a larger effect on CVD risk in the groups with diets low or average for fresh vegetables, fruits, and berries (hazard ratio [HR] = 1.22, *p* = 3.0×10^−4^, and HR = 1.35, *p* = 4.1×10^−3^, respectively) compared to the group with high consumption of these foods (HR = 0.96, *p* = 0.73) (*p* = 0.0011 for difference). The combination of the least prudent diet and two copies of the risk allele was associated with a 2-fold increase in risk for MI (OR = 1.98, *p* = 2.11×10^−9^) in the INTERHEART study and a 1.66-fold increase in risk for CVD in the FINRISK study (HR = 1.66, *p* = 0.0026).

**Conclusions:**

The risk of MI and CVD conferred by Chromosome 9p21 SNPs appears to be modified by a prudent diet high in raw vegetables and fruits.

*Please see later in the article for the Editors' Summary*

## Abstract 2

Ron Do and colleagues find that a prudent diet high in raw vegetables may modify the increased genetic risk of cardiovascular disease conferred by the chromosome 9p21 SNP.

## Editors' Summary

**Background:**

Cardiovascular diseases (CVDs)—diseases that affect the heart and/or the blood vessels—are a leading cause of illness and death worldwide. In the United States, for example, the leading cause of death is coronary heart disease, a CVD in which narrowing of the heart's blood vessels by fatty deposits slows the blood supply to the heart and may eventually cause a heart attack (myocardial infarction, or MI); the third leading cause of death in the US is stroke, a CVD in which the brain's blood supply is interrupted. Environmental factors such as diet, physical activity, and smoking alter a person's risk of developing CVD. In addition, certain genetic variants (alterations in the DNA that forms the body's blueprint; DNA is packed into structures called chromosomes) alter the risk of developing CVD and are passed from parent to child. Thus, in CVD, as in most common diseases, both genetics and the environment play a role.

**Why Was This Study Done?:**

Recent studies have identified several genetic variants that are associated with an increased risk of developing CVD. One of the most robust of these genetic associations is a cluster of single nucleotide polymorphisms (SNPs, differences in a single DNA building block) in a chromosomal region (locus) called 9p21. So far, this association has been mainly studied in European populations. Moreover, the interaction of this locus with environmental factors has not been extensively studied. A better understanding of how 9p21 variants affect CVD risk in people of different ethnicities and of the interaction between this locus and environmental factors could allow the development of targeted strategies for the prevention of CVD. In this study, the researchers investigate the association of 9p21 risk variants with CVD in people of different ethnicities and test for an interaction between this locus and environmental factors.

**What Did the Researchers Do and Find?:**

The researchers assessed four 9p21 SNPs in people enrolled in the INTERHEART study, a global retrospective case-control study that investigated potential MI risk factors by comparing people who had had an acute non-fatal MI with similar people without heart disease. All four SNP risk variants increased the risk of MI by about a fifth. However, the effect of the SNPs on MI was influenced by the “prudent” diet pattern score of the INTERHEART participants, a score that includes fresh fruit and vegetable intake as recorded in food frequency questionnaires. That is, the risk of MI in people carrying SNP risk variants was influenced by their diet. The strongest interaction was seen with an SNP called rs2383206, but although rs2383206 carriers who ate a diet poor in fruits and vegetables had a higher risk of MI than people with a similar diet who did not carry this SNP, rs2383206 carriers and non-carriers who ate a fruit- and vegetable-rich diet had a comparable MI risk. Overall, the combination of the least “prudent” diet and two copies of the risk variant (human cells contain two complete sets of chromosomes) was associated with a two-fold increase in risk for MI in the INTERHEART study. Additionally, data collected in the FINRISK study, which characterized healthy individuals living in Finland at baseline and then followed them to see whether they developed CVD, revealed a similar interaction between diet and 9p21 SNPs.

**What Do These Findings Mean?:**

These findings suggest that the risk of CVD conferred by chromosome 9p21 SNPs may be influenced by diet in multiple ethnic groups. Importantly, they suggest that the deleterious effect of 9p21 SNPs on CVD might be mitigated by consuming a diet rich in fresh fruits and vegetables. The accuracy of these findings may be affected by recall bias in the INTERHEART study (that is, some people may not have remembered their diet accurately) and by the small number of CVD cases in the FINRISK study. Nevertheless, these findings suggest that gene–environment interactions are important drivers of CVD, and they raise the possibility that a sound diet can mediate the effects of 9p21 SNPs.

**Additional Information:**

Please access these websites via the online version of this summary at http://dx.doi.org/10.1371/journal.pmed.1001106.

- The American Heart Association provides information about many types of cardiovascular disease for patients, caregivers, and professionals and tips on keeping the heart healthy
- The UK National Health Service Choices website provides information about cardiovascular disease and stroke
- Information is available from the British Heart Foundation on heart disease and keeping the heart healthy
- The US National Heart Lung and Blood Institute provides information on a wide range of cardiovascular diseases
- MedlinePlus provides links to many other sources of information on heart diseases, vascular diseases, and stroke (in English and Spanish)
- The US Centers for Disease Control and Prevention has a simple fact sheet on gene-environment interactions; the US National Institute of Environmental Health Sciences provides links to other information on gene-environment interactions
- More information is available on the INTERHEART study and on the FINRISK study

## Introduction

Cardiovascular disease (CVD), including myocardial infarction (MI), is one of the leading causes of death and disability worldwide [1]. The etiology of the disease is well characterized and includes environmental and behavioral factors such as diet, physical activity, smoking, and alcohol consumption [2]. A familial component also exists for the disease; a stronger concordance for coronary heart disease is found in monozygotic twins than in dizygotic twins [3], and family history is an independent risk factor for cardiovascular events [4]. Recent genome-wide association studies have identified genetic variants in the Chromosome 9p21 region that are significantly associated with CVD [5],[6] and MI [7]. These findings have been replicated mostly in European studies [5]–[9], but also in East Asian [10] and Hispanic [11] populations. Subsequent reports have demonstrated that the locus is also associated with abdominal aortic aneurysms, intracranial aneurysms [12], and periodontal disease [13].

Variation in the effect of genetic factors may be partly attributed to varying levels of environmental exposures such as physical activity, smoking, and diet (see [14] for a recent review). In particular, diet plays an important role in the development of heart disease, with high consumption of trans fats and high glycemic carbohydrates, and low consumption of fruits, vegetables, fish, nuts, and whole grains, being associated with MI in observational studies [15], and with changes in diet clearly modifying CVD outcomes [15]–[17]. Despite recent progress in identifying some novel genetic contributors to CVD, it is currently unknown how these recently discovered loci interact with the environment and what role such interactions play in the development of disease [14],[18]. Investigation of gene–environment interactions are necessary to further our understanding of the underlying biology and pathophysiology of the disease [19], and could potentially be useful in improving cardiovascular risk stratification [18] and thereby reducing clinical events [18],[20]. Gene–environment interaction studies could also contribute to explaining some of the phenotypic variance that is not accounted for by common variants [21].

Previous gene–environment interaction studies have largely been plagued by small sample sizes and hence reduced power, and have been mostly limited to single environmentally homogenous populations of European origin. In the present study, we conducted a large-scale multiethnic study of variants in the 9p21 region and acute MI in 8,114 individuals (3,820 cases and 4,294 controls) from five ethnicities—European, South Asian, Chinese, Latin American, and Arab—who participated in the INTERHEART study [2]. We first confirm the association of 9p21 with MI/CVD in multiple ethnicities and then examine the role of environmental exposures in influencing the magnitude of these genetic associations. We then follow up our findings in 19,129 Finnish individuals with 1,014 incident cases of CVD from the prospective FINRISK Study.

## Materials and Methods

Individuals from five ethnicities participating in the INTERHEART study were genotyped for the present investigation. INTERHEART is a standardized global retrospective case-control study of risk factors for acute non-fatal MI [2]. Cases were recruited within 24 h of being admitted to a coronary care unit or cardiology ward with clinical characteristics of acute MI. Controls matched for age and sex and without a history of heart disease symptoms were recruited from the hospital or the community. The study was conducted between 13 February 1999 and 11 November 2003. Standardized dietary phenotypes were measured by a short qualitative food frequency questionnaire (FFQ) of 19 food items. Specific food pattern scores were derived from the food items using factor analysis. Factors were orthogonally rotated (varimax) to generate uncorrelated factors. Three factors (dietary patterns) were retained based on criteria including an eigenvalue >1.0 and scree plot, and were subjectively labeled as oriental (soy sauce, tofu, pickled foods, green leafy vegetables, eggs, and low sugar), western (eggs, meats, fried and salty foods, sugar, nuts, and desserts), and prudent (raw vegetables, fruits, green leafy vegetables, nuts, desserts, and dairy products) based on the food items retained for each factor (which had factor loadings >0.25) as described in [22]. Raw vegetable intake and fruit intake had the highest factor loadings for the prudent diet score and therefore made up the largest components of the score [22]. A dietary risk score was also generated, using a point system from the food items [22] that included a tabulation of food items that were considered to be protective of CVD (fruits and green leafy vegetables, other cooked vegetables, and other raw vegetables) and associated with risk (meat, salty snacks, and fried foods). In the present 9p21 study, the prudent diet was protective against MI (OR = 0.81, 95% CI 0.77–0.85), the western diet pattern was associated with an increased risk of MI (OR = 1.14, 1.09–1.19), and the oriental diet pattern was not associated with MI (OR = 0.94, 0.88–1.06) [22]. Further details on case and control recruitment as well as the measurement of specific phenotypes for the INTERHEART study can be obtained from [2] and [22].

The FINRISK study is a series of population-based CVD risk factor surveys conducted every 5 y in Finland [23]. CVD was defined as coronary deaths, non-fatal MI, unstable angina, revascularization (coronary artery bypass graft or percutaneous transluminal coronary angioplasty), and ischemic stroke events. Surveys conducted in 1992, 1997, and 2002 were included in the present analysis. Dietary information was collected from a FFQ consisting of up to 130 food items. A composite score based on three responses to questions about fruit, vegetable, and berry intake was created. This composite category has been shown to be associated with CVD-related events [24]. Diet groups with low, medium, and high consumption of vegetables, fruits, and berries were defined by the number of positive responses to the questions, how often do you eat (1) fresh vegetables; (2) fresh fruits; (3) fresh or frozen berries? Individuals who chose “daily or several times a day” for at least two of these three questions were assigned to the diet group “high consumption” or “prudent”; individuals who chose “daily or several times a day” for one question and “almost every day” for another question were assigned to the diet group “medium consumption”; and all other individuals were assigned to the diet group “low consumption.”

For the INTERHEART samples, four SNPs (rs10757274, rs2383206, rs10757278, rs1333049) from the Chromosome 9p21 region were selected based on previous results from genome-wide association studies for coronary heart disease/MI [5]–[7]. Genotyping was performed using the Sequenom iPLEX Gold Assay, except for the Chinese samples, where genotypes were produced using the Illumina GoldenGate genotyping assay and the BeadStudio software package. All genotyped SNPs had a high call rate with both technologies (>98%). Deviation from Hardy-Weinberg equilibrium was assessed by analyzing the genotypic distributions in controls only, using an exact test within each of the five ethnicities. All SNPs were in Hardy-Weinberg equilibrium in the controls of each ethnicity (*n* = 20 tests) except for rs2383206 in Arabs (*p* = 0.02) [25]. Genotypes were successfully produced for 1,744 Europeans, 1,867 South Asians, 2,231 Chinese, 1,100 Latin Americans, and 1,172 Arabs (a total of 8,114 samples). For the FINRISK study, rs4977574 from the 9p21 region had been previously genotyped using the Sequenom iPLEX Gold Assay. This SNP was selected because it is in high linkage disequilibrium (LD) with rs2383206 (*r* ^2^ = 0.91) in HapMap Europeans. We analyzed the genotypes for 19,129 FINRISK individuals (including 1,014 incident cases of CVD).

Association testing within each ethnicity in the INTERHEART samples was performed using logistic regression, and association analysis of all individuals was performed using the Cochran-Mantel-Haenszel test. Individuals with missing values for either SNPs or environmental factors were excluded from the analyses (*n*∼200). We tested for interactions between 9p21 SNPs and physical activity, smoking, and previously defined diet variables (INTERHEART dietary risk score, prudent diet, oriental diet, and western diet) for a total of six independent tests. Once an interaction was found, we performed a secondary analysis on variables that were closely related to the primary variables (based on high factor loadings) and were previously shown to be associated with MI [22]. Adjustment for multiple testing using the Bonferroni correction was based on the six primary tests and does not include variables tested in the secondary analysis, because of the strong correlations between the primary and secondary variables.

Interaction analyses were performed by including the main effects of the SNP and the environmental variable (physical activity, smoking, or diet) in addition to the interaction term in the model after adjusting for ethnicity. In the stratified association analysis of 9p21 by prudent diet score in all ethnicities combined, tertiles were determined for each ethnicity and then grouped together into low, medium, and high prudent pattern score classes. Within each of these classes, association testing of 9p21 and MI was performed using logistic regression after adjusting for ethnicity. Tests of homogeneity of effect size between the different prudent diet classes were performed using the Breslow-Day test on the 9p21 alleles. Joint effects of all combinations of 9p21 genotype and groups of prudent diet score were compared to the reference group of individuals bearing two copies of the protective allele and a high prudent diet score using logistic regression after adjusting for ethnicity. For the FINRISK study, Cox proportional hazard models, adjusted for age and sex, were used for estimating the effects of 9p21 and diet groups on CVD. Interaction analyses were performed by including the main effects of the SNP and diet in addition to the interaction term in the model after adjusting for study area (eastern versus southwestern Finland).

All analyses were performed using SAS (version 8.2; SAS Institute) or PLINK [26]. LD maps and *r* ^2^ values were produced using Haploview [27]. All reported *p*-values are unadjusted for multiple testing unless specified.

The INTERHEART and FINRISK studies were approved by institutional review boards. Informed consent was obtained from all participants, and all work was conducted according to the principles expressed in the Declaration of Helsinki.

## Results

Demographic characteristics of the INTERHEART participants included in this analysis are presented in Table S1. They are consistent with what has been previously reported for the study [28]. For this genetic study, 3,820 cases and 4,294 controls from the INTERHEART study were analyzed. These samples represent 27% of the entire study and were selected from the ethnicities with the largest representation.

Allele frequencies varied from 0.48 to 0.66 for the four genotyped SNPs in the five ethnic groups (Table 1) of the INTERHEART samples, and the SNPs were in high LD in Europeans, South Asians, and Chinese (all *r* ^2^≥0.80), with more moderate LD observed in Latin Americans and Arabs (Figure S1). The four 9p21 SNPs were associated with MI in the combined sample that included all ethnicities, with ORs ranging from 1.18 to 1.20, and *p*-values ranging from 1.85×10^−8^ to 5.21×10^−7^ (Table 2). All four SNPs were significantly associated in Europeans (1.17≤OR≤1.18, 0.016≤*p*≤0.024), South Asians (1.22≤OR≤1.27, 0.0003≤*p*≤0.0025), and Chinese (1.16≤OR≤1.21, 0.0017≤*p*≤0.011), and three were associated in Latin Americans (1.22≤OR≤1.32, 0.0066≤*p*≤0.029). None of the SNPs demonstrated a significant association with MI in Arabs, although the direction of effect for all four SNPs was consistent with that of the other ethnicities (1.04≤OR≤1.12). Of the 9p21 associations, the most significant were for rs2383206 and rs1333049 in South Asians (both *p* = 0.0003).

**Table 1:** Allele frequencies of SNPs by ethnicity.

SNP	+/−	European	South Asian	Chinese	Arab	Latin American
rs10757274	C/T	0.50	0.54	0.48	0.56	0.50
rs2383206	G/A	0.53	0.57	0.49	0.66	0.53
rs10757278	C/T	0.50	0.54	0.50	0.48	0.50
rs1333049	C/G	0.49	0.53	0.50	0.48	0.49

Allele frequencies of the risk allele (+) are shown.

**Table 2:** Association results of Chromosome 9p21 SNPs and acute myocardial infarction.

SNP	+/−	All Individuals^a^		European		South Asian		Chinese		Latin American		Arab	
		OR (CI)	*p*-Value	OR (CI)	*p*-Value	OR (CI)	*p*-Value	OR (CI)	*p*-Value	OR (CI)	*p*-Value	OR (CI)	*p*-Value
rs10757274	C/T	1.19 (1.12–1.27)	**3.07E-08**	1.18 (1.03–1.35)	**0.017**	1.26 (1.11–1.40)	**0.0008**	1.21(1.07–1.36)	**0.0017**	1.22 (1.02–1.45)	**0.029**	1.05 (0.89–1.23)	0.58
rs2383206	G/A	1.18 (1.10–1.25)	**5.21E-07**	1.18 (1.03–1.35)	**0.018**	1.25 (1.10–1.40)	**0.0003**	1.17 (1.04–1.31)	**0.010**	1.13(0.94–1.35)	0.19	1.10 (0.93–1.30)	0.27
rs10757278	C/T	1.20 (1.12–1.27)	**1.85E-08**	1.18 (1.03–1.35)	**0.016**	1.22 (1.07–1.40)	**0.0025**	1.17 (1.04–1.31)	**0.0086**	1.32(1.11–1.58)	**0.002**	1.12 (0.95–1.31)	0.18
rs1333049	C/G	1.18 (1.11–1.26)	**8.51E-08**	1.17 (1.02–1.34)	**0.024**	1.27 (1.12–1.40)	**0.0003**	1.16 (1.04–1.31)	**0.011**	1.28 (1.07–1.52)	**0.0066**	1.04 (0.89–1.22)	0.59

+ is the risk allele; associations with *p*<0.05 are shown in bold. Results shown are unadjusted ORs and *p*-values.

a Cochran-Mantel-Haenszel test.

We next examined whether physical activity, smoking, or diet influenced the effect of chromosome 9p21 SNPs on MI in the INTERHEART study. No significant interaction or trend was found with physical activity (OR = 0.90, *p* = 0.23) or smoking (OR = 0.98, *p* = 0.56). Dietary interaction was investigated by testing the INTERHEART dietary risk score and the three food pattern scores derived from factor analysis [22] (see Methods). No significant interactions were found for the dietary risk score or for the oriental diet score, while the SNP rs2383206 had only a nominal interaction with the western diet score (*p* = 0.028). However, significant interactions with the prudent diet score were found for all four SNPs for the outcome of MI (Table 3), with the strongest effect seen for the interaction of rs2383206 (unadjusted *p* = 0.0004, and adjusted for the risk factors apoB/apoA1, waist/hip ratio, diabetes, hypertension, and smoking, *p* = 0.018). We also tested individual food items that were closely related to the three factors (based on high factor loadings) that were previously shown to be associated with MI. The strongest interactions for all four SNPs were with raw vegetable intake (all *p*<0.008) (Table 3).

**Table 3:** The effect of the interaction of diet and Chromosome 9p21 SNPs on acute myocardial infarction.

SNP	Dietary Risk Score	Food Pattern			Intake of Individual Food Items								
		Oriental	Western	Prudent	Meat/Poultry	Whole Grains	Refined Grains	Deep-Fried Foods	Salty Foods	Fruits	Green Leafy Vegetables	Raw Vegetables	Cooked Vegetables
rs10757274	0.13	0.64	0.19	**0.0017**	0.87	0.60	0.38	0.62	0.056	0.48	0.70	**0.0055**	0.51
rs2383206	0.14	0.86	**0.028**	**0.0004**	0.93	0.53	0.98	0.87	**0.012**	0.28	0.96	**0.0014**	0.21
rs10757278	0.25	0.74	0.11	**0.0022**	0.97	0.75	0.44	0.96	**0.022**	0.47	0.81	**0.0071**	0.25
rs1333049	0.24	0.92	0.17	**0.0011**	0.97	0.36	0.52	0.98	**0.038**	0.36	0.85	**0.0041**	0.48

Interaction tests were performed using logistic regression. Interaction term *p*-values are shown, after adjustment for main effects of SNP and the dietary variable, as well as ethnicity. The minimum sample size for the analyses is *n* = 7,989. Associations with *p*<0.05 are shown in bold. Included individual food items were closely related to the three factors (based on high factor loadings) and were previously shown to be associated with MI.

The interaction of rs2383206 and prudent diet under an unadjusted model remained statistically significant after correcting for six independent tests (physical activity, smoking, dietary risk score, western diet score, oriental diet score, prudent diet score) using a Bonferroni correction (*p* = 0.0004×6 = 0.0024). But after adjustment for raw vegetable intake and its interaction with rs2383206, the interaction of the SNP with prudent diet was markedly diminished (*p* = 0.047 for the interaction term). The interaction of this SNP and prudent diet was not markedly diminished after an adjustment for each of the other major components of the prudent diet [22], including dairy intake (*p* = 0.0066 for the interaction of SNP and prudent diet), nut intake (*p* = 0.0008), green leafy vegetable intake (*p* = 0.0002), fruit intake (*p* = 0.0015), and dessert intake (*p* = 0.0094).

**Figure 1:**
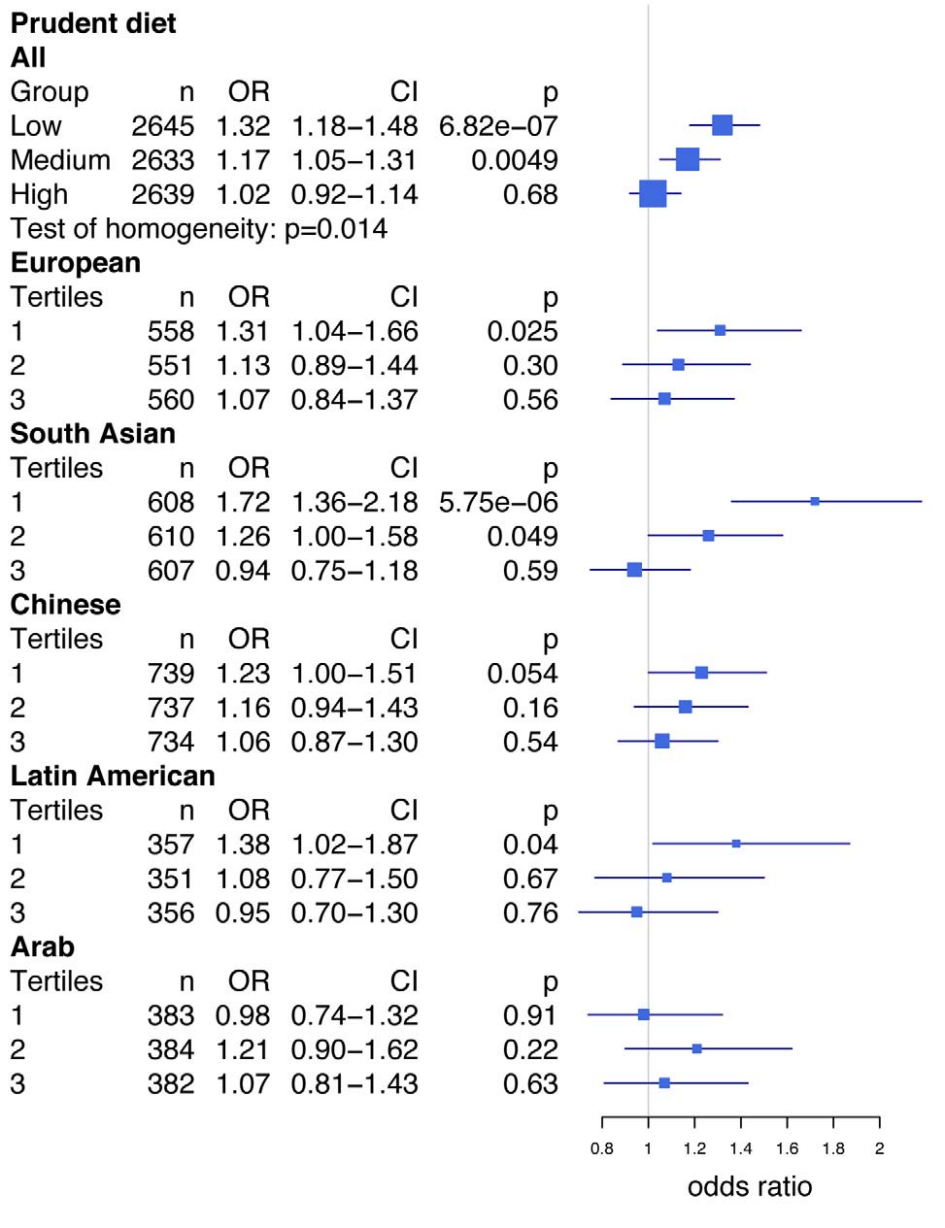
Prudent diet modifies the effect of the Chromosome 9p21 variant rs2383206 on acute myocardial infarction in the INTERHEART study. Prudent diet is derived from a factor analysis and is made up of several individual food items, including fruits and raw vegetables. The test of homogeneity was performed using the Breslow-Day test. ORs and 95% confidence intervals are shown in blue. ORs presented are per allele, i.e., the OR equals the increase in risk for each copy of the risk allele. Box sizes are proportional to the precision of the estimate (1/standard error^2^). Tertiles for prudent diet (1, 2, and 3, with 1 being the lowest prudent diet score group) were calculated for each ethnicity separately.

A closer examination of this interaction revealed that rs2383206 was strongly associated with MI in the group with the lowest prudent diet score (Figure 1; first tertile, OR = 1.32, 95% CI 1.18–1.48, *p* = 6.82×10^−7^ for all individuals), while the effect was diminished in a step-wise fashion for the medium (second tertile, OR = 1.17, 95% CI 1.05–1.31, *p* = 0.0049 for all individuals) and high scoring prudent diet groups (third tertile, OR = 1.023, 95% CI 0.92–1.14, *p* = 0.68 for all individuals). The interaction was strongest in South Asians and Latin Americans (Figure 1). The Breslow-Day test revealed heterogeneity for the allelic association of rs2383206 with MI between the three groups (low, medium, and high) of the prudent diet score (chi^2^ = 8.55, *p* = 0.014; Figure 1). Similar results were found with raw vegetable intake (data not shown).

**Figure 2:**
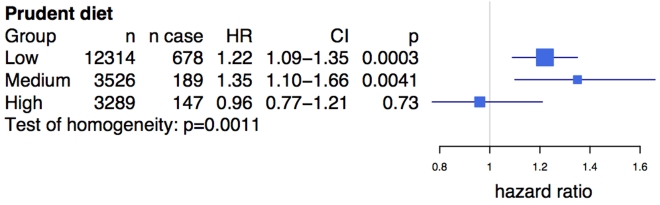
Prudent diet modifies the effect of the Chromosome 9p21 variant rs4977574 on cardiovascular disease in the FINRISK study. Prudent diet is derived from a composite score of fruit, vegetable, and berry intake (see Methods). The test of homogeneity was performed using the Breslow-Day test. HRs and 95% confidence intervals are shown in blue. HRs presented are per allele. Box sizes are proportional to the precision of the estimate (1/standard error^2^).

We observed a similar diet interaction using a related composite dietary variable (see Methods) in the prospective FINRISK study, consisting of 19,129 Finnish individuals with 1,014 incident cases of CVD. The average age of participants in the study was 46.7 y, and 55% were female (Table S2). rs4977574, a strong proxy SNP for rs2383206 (see Methods), was associated with CVD (HR = 1.17, 95% CI 1.075–1.275, *p* = 0.0003). A diet high in vegetables, fruits, and berries was inversely associated with CVD (HR = 0.79, 95% CI 0.66–0.94, *p* = 0.0076), unlike the diet lowest in these food groups. Similar to the findings from the INTERHEART analysis, the Chromosome 9p21 SNP showed an effect of the risk allele on incident CVD among individuals with low (HR = 1.22, *p* = 3×10^−4^) and medium (HR = 1.35, *p* = 4.1×10^−3^) consumption of vegetables, fruits, and berries, but demonstrated no effect in the high consumption group (HR = 0.96, *p* = 0.73) (Figure 2). Differences in the effect size between the different diet classes were statistically significant (test of homogeneity *p* = 0.0011).

**Figure 3:**
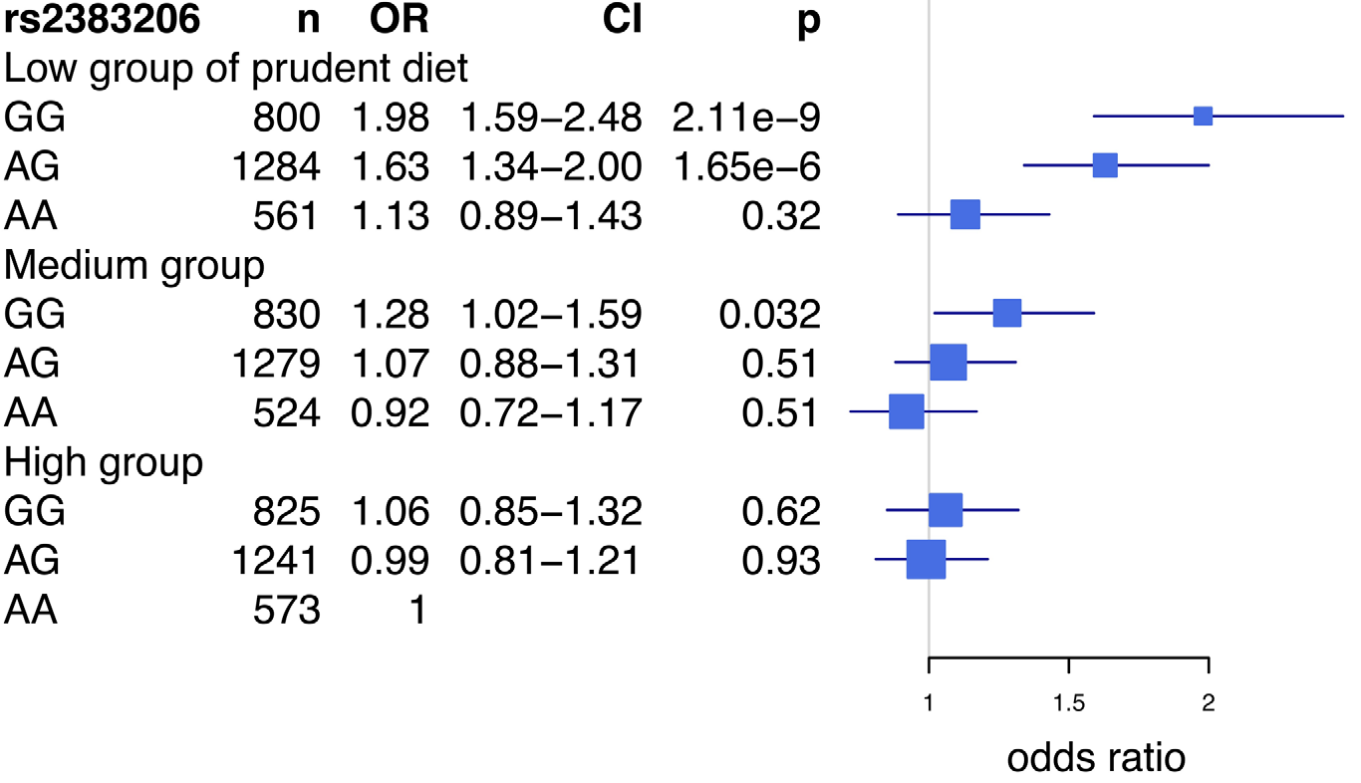
Risk of acute myocardial infarction associated with prudent diet and the Chromosome 9p21 variant rs2383206 in the INTERHEART study. The reference class is the group bearing two protective alleles for rs2383206 (genotype AA) and having a high prudent dietary pattern score. ORs and 95% confidence intervals are shown in blue. Box sizes are proportional to the precision of the estimate (1/standard error^2^).

**Figure 4:**
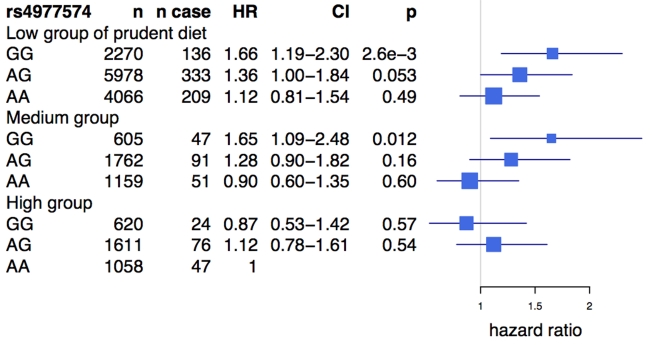
Risk of cardiovascular disease associated with prudent diet and the Chromosome 9p21 variant rs4977574 in the FINRISK study. The reference class is the group bearing two protective alleles for rs4977574 (genotype AA) and having a prudent diet (see Methods). HRs and 95% confidence intervals are shown in blue. Box sizes are proportional to the precision of the estimate (1/standard error^2^).

An analysis of the rs2383206 genotype and tertiles of the prudent diet score in the INTERHEART samples demonstrated that individuals with two copies of the risk allele and with a low prudent diet score had a ∼2-fold increase in MI risk when compared to the reference group of individuals with two copies of the protective allele and a high prudent diet score (Figure 3). An increased risk was also observed with other groups, but in a diminishing step-wise fashion (Figure 3). In particular, for the group with the most prudent diet, very little effect was observed for 9p21 variation. Similarly, in the FINRISK study, the combination of a diet low in fruits, berries, and vegetables and two copies of the risk allele was associated with a 1.66-fold increase in risk for CVD (HR = 1.66, *p* = 0.003) (Figure 4), but again no consistent effect of the 9p21 SNP was observed in the group with the most prudent diet.

## Discussion

We report that dietary intake may influence the effect of Chromosome 9p21 SNPs on MI and CVD in multiple ethnic groups of the INTERHEART study and in FINRISK, a large prospective study. We demonstrate the consistent effect of 9p21 SNPs on MI and CVD among individuals who have a low prudent diet score, while the effect is diminished among individuals who consume a more prudent diet. In particular, in the individuals who are homozygous for the risk allele of rs2383206 and have a low prudent diet score, we observe a 1.6- to 2.0-fold increase in the risk for MI when compared to the reference group.

Consistent with previous results, we demonstrate that 9p21 variants influence MI in many populations, but the risk may vary across populations. In the present study, we observed ORs ranging from 1.10 in Arabs to 1.25 in South Asians for rs2383206. While such differences could be due to chance, they could also be due to differential LD with a causative SNP, or to environmental exposures. The strongest interaction of 9p21 SNPs was found in Latin Americans and South Asians, the same ethnic groups that had stronger protective effects of fruit and vegetable consumption in the INTERHEART study [29],[30]. One SNP was not associated with MI in Latin Americans, and no association of any 9p21 SNP with MI was significant in the Arab samples. However, the direction of effect of the risk alleles was always consistent with the significant effects observed in other ethnic groups. The non-significant results in the Latin American and Arab groups could be due to a lack of power, as these study populations contained the smallest number of individuals (*n*<1,172) compared to the other ethnic groups. It could also be due to lower LD with an unidentified causal SNP, as the LD across the genotyped SNPs is reduced in both the Latin American and Arab samples, while the LD was higher among the other ethnic groups (Figure S1).

The interaction of 9p21 SNPs with diet is intriguing because very little is known about how these variants influence CVD. 9p21 variants are not associated with known risk factors for CVD [5],[31],[32], but some studies have implicated them in vascular structure [12],[33]. Doria et al. [34] report an interaction of 9p21 and glycosylated hemoglobin, a marker of hyperglycemia, on coronary heart disease in individuals with type 2 diabetes. They observed that the risk of 9p21 was magnified in the presence of poor glycemic control. While the mechanism of this interaction is unknown, further research is warranted to investigate whether the increased effect of 9p21 in type 2 diabetics with poor glycemic control is mediated through diet. However, one study that identified a dietary pattern very similar to the INTERHEART prudent score, characterized by high intake of fruits and vegetables, pasta, rice, fish, and dairy, showed that this diet was inversely associated with fasting glucose and the metabolic syndrome [35]. In addition, Visel et al. [36] have shown that targeted deletion of the non-coding region of 9p21 influences the expression of neighboring genes in cardiac tissue and increases mortality in mice on a high fat, high cholesterol diet when compared to wild-type mice.

Limitations of this study include the differences in the 9p21 SNP, the cardiovascular outcome, and the dietary variables between the INTERHEART and FINRISK studies. The 9p21 SNPs used in the studies are in the same LD block and have an *r* ^2^ = 0.91 and hence should lead to consistent results between the studies. In addition, CVD was the outcome used in the FINRISK study, rather than MI, to increase the number of events and hence statistical power. However, the genetic association of 9p21 and CVD is well documented, and our results with CVD suggest that the effect impacts the composite CVD phenotype.

The difference in dietary variables used between the two studies highlights a challenge for all gene–environment interaction studies that rely on replication studies. It remains difficult to obtain independent cohorts that have identical environmental exposure data, especially for diet. Our analysis in the INTERHEART case-control study was based on a factor (Factor 3: prudent food pattern) derived from a qualitative FFQ. Our results were analyzed as a continuous trait but presented as tertiles for clarity of presentation. Because a different FFQ was used in the FINRISK study, we used a combined variable consisting of fruit, vegetable, and berry intake in the FINRISK study as the closest proxy to the prudent diet score used in the INTERHEART study, since fruit and raw vegetable intake have the highest factor loadings for the prudent diet score (see Methods). This resulted in three unequal groups, as the majority of FINRISK individuals had less than daily consumption of all three food groups. While fruit, vegetable, and berry intake was associated with a reduced incidence of CVD in the FINRISK study (consistent with the INTERHEART results), the group with the highest intake, which demonstrated no effect of the 9p21 variants, contained only 147 CVD cases, and thus these data should be interpreted with caution.

Recall bias in dietary intake measurement is more likely to occur in cases compared to controls, as acute illness may lead to underreporting or overreporting of consumption of various food items. However, the dietary associations described in the INTERHEART study are consistent with other reports of dietary components associated with CVD [15], thus increasing the validity of the dietary questionnaire that was used. In addition, a prospective study such as FINRISK in which dietary history is collected prior to a CVD event minimizes the chance of recall bias.

Another limitation of our investigation is that we have observed consistent results in only one case-control and one prospective study. Despite the discrepancies in SNP, dietary variable, and outcomes, the fact that our gene–environment interaction results are consistent between the INTERHEART and FINRISK studies suggests that our results may be robust. In addition, in the INTERHEART samples we observed a consistent trend of increasing effect size for the 9p21 MI risk allele across decreasing prudent diet tertiles not only in all individuals, but also in multiple ethnic groups analyzed separately (Figure 1). Future research should include reproducing this study approach in large study samples that have similar genetic and dietary information.

We have observed the 9p21 and diet interaction in a case-control study as well as a prospective cohort study (FINRISK). To date, the present work is one of the largest gene–diet interaction studies of CVD ever conducted (*n* = 27,243). Our findings suggest that there may be an important interplay of genes and environment in the etiology of CVD, and could shed light on the underlying pathophysiology of 9p21.

## Supporting Information

Figure S1 **Linkage disequilibrium of four Chromosome 9p21 SNPs in Europeans, South Asians, Chinese, Latin Americans, and Arabs in the INTERHEART study.** Numbers and shades of grey indicate *r* ^2^ values.
(TIF)

Table S1 **Characteristics of the INTERHEART participants.** All values are means (standard deviation) unless otherwise specified. s/day, servings per day. 3,820 cases (47.1%) and 4,294 controls (52.9%) were analyzed in the present study. Only individual food items investigated in this study are shown.
(DOC)

Table S2 **Characteristics of the FINRISK participants.** All values are proportions unless otherwise specified.
(DOC)

Text S1 **INTERHEART investigators.**
(DOC)

## Abbreviations

CVD: cardiovascular disease
FFQ: food frequency questionnaire
HR: hazard ratio
LD: linkage disequilibrium
MI: myocardial infarction
OR: odds ratio
