# Supplementary figures and images for: The Effect of Chromosome 9p21 Variants on Cardiovascular Disease May Be Modified by Dietary Intake: Evidence from a Case/Control and a Prospective Study
Source: PLoS Med. 2011 Oct 11;8(10):e1001106. doi: 10.1371/journal.pmed.1001106 (PMC3191151; doi:10.1371/journal.pmed.1001106)

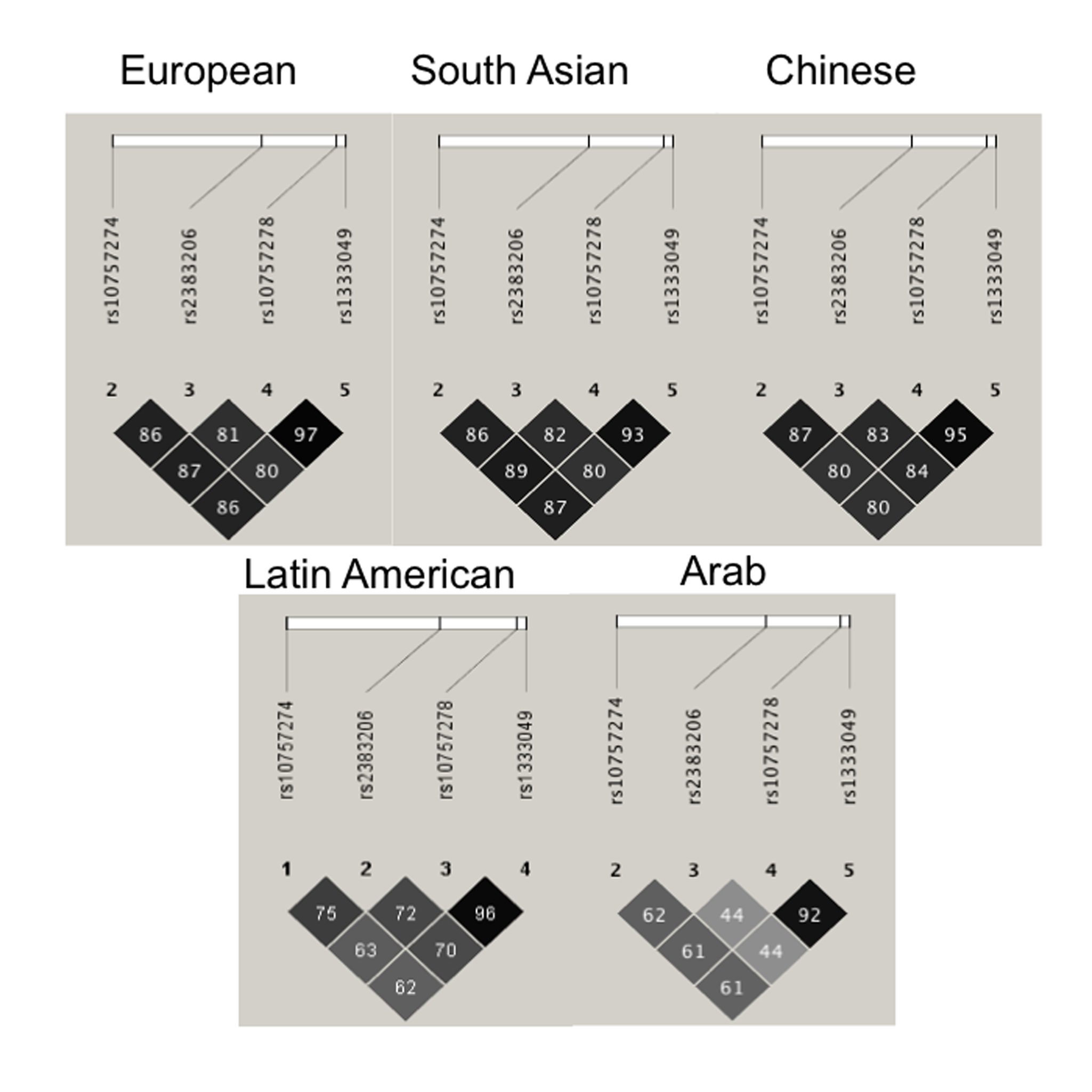

Supplement: Figure S1 — Linkage disequilibrium of four Chromosome 9p21 SNPs in Europeans, South Asians, Chinese, Latin Americans, and Arabs in the INTERHEART study. Numbers and shades of grey indicate r 2 values. (TIF) [file pmed.1001106.s001.tif]
