# Supplementary material for: The Effect of Chromosome 9p21 Variants on Cardiovascular Disease May Be Modified by Dietary Intake: Evidence from a Case/Control and a Prospective Study
Source: PLoS Med. 2011 Oct 11;8(10):e1001106. doi: 10.1371/journal.pmed.1001106 (PMC3191151; doi:10.1371/journal.pmed.1001106)
Supplement: Table S1 — Characteristics of the INTERHEART participants. All values are means (standard deviation) unless otherwise specified. s/day, servings per day. 3,820 cases (47.1%) and 4,294 controls (52.9%) were analyzed in the present study. Only individual food items investigated in this study are shown. (DOC) [file pmed.1001106.s002.doc]

Table S1. Demographic, physical and dietary characteristics of the INTERHEART subjects

|  | European | N | South Asian | n | Chinese | n | Latin American | n | Arab | n |
| --- | --- | --- | --- | --- | --- | --- | --- | --- | --- | --- |
| Case status , n (%) | 829 (47.53) | 1744 | 941 (50.40) | 1867 | 1124 (50.38) | 2231 | 374 (34.00) | 1100 | 552 (47.10) | 1172 |
| Sex (female), n (%) | 529 (30.33) | 1744 | 186 (9.96) | 1867 | 1587 (71.13) | 2231 | 255 (23.18) | 1100 | 138 (11.77) | 1172 |
| Age (years) | 61.45 (12.30) | 1744 | 50.42 (10.73) | 1867 | 59.31 (10.55) | 2231 | 59.63 (12.69) | 1100 | 50.77 (9.34) | 1172 |
| Physical activity, n (%) | 521 (30.15) | 1728 | 109 (5.97) | 1825 | 205 (9.20) | 2228 | 234 (21.65) | 1081 | 86 (7.39) | 1164 |
| Dietary risk score | -6.06 (5.26) | 1724 | -4.56 (4.79) | 1857 | -3.38 (4.99) | 2224 | -6.37 (5.93) | 1093 | -5.43 (4.49) | 1166 |
| Meat/poultry (s/day) | 0.75 (0.58) | 1741 | 0.42 (0.50) | 1862 | 0.66 (0.52) | 2229 | 0.68 (0.58) | 1098 | 0.48 (0.32) | 1172 |
| Whole grains (s/day) | 0.56 (0.80) | 1744 | 1.43 (1.28) | 1867 | 0.64 (0.80) | 2230 | 0.57 (0.75) | 1100 | 0.63 (1.078) | 1172 |
| Refined grains (s/day) | 0.81 (0.99) | 1744 | 0.96 (0.99) | 1867 | 1.59 (1.064) | 2231 | 1.19 (1.34) | 1100 | 1.17 (1.019) | 1172 |
| Deep fried foods (s/day) | 0.083 (0.20) | 1741 | 0.15 (0.30) | 1867 | 0.16 (0.24) | 2231 | 0.17 (0.39) | 1100 | 0.11 (0.24) | 1171 |
| Salty foods (s/day) | 0.24 (0.58) | 1743 | 0.26 (0.63) | 1866 | 0.39 (0.59) | 2230 | 0.12 (0.41) | 1100 | 0.27 (0.79) | 1171 |
| Fruits (s/day) | 1.17 (0.98) | 1742 | 0.50 (0.55) | 1867 | 0.61 (0.52) | 2231 | 0.91 (0.85) | 1099 | 0.75 (0.62) | 1171 |
| Green leafy vegetables (s/day) | 0.48 (0.48) | 1737 | 0.54 (0.56) | 1864 | 1.19 (0.80) | 2230 | 0.71 (0.56) | 1097 | 0.59 (0.56) | 1171 |
| Other raw vegetables (s/day) | 0.43 (0.44) | 1736 | 0.37 (0.48) | 1865 | 0.26 (0.32) | 2230 | 0.45 (0.46) | 1097 | 0.43 (0.44) | 1172 |
| Other cooked vegetables (s/day) | 0.49 (0.43) | 1740 | 0.83 (0.78) | 1864 | 0.92 (0.98) | 2228 | 0.58 (0.54) | 1099 | 0.39 (0.33) | 1170 |
| Oriental dietary pattern score | -0.50 (0.51) | 1699 | -0.42 (0.58) | 1838 | 1.10 (0.89) | 2213 | -0.57 (0.48) | 1086 | -0.37 (0.54) | 1156 |
| Western dietary pattern score | -0.17 (0.91) | 1699 | -0.16 (0.90) | 1838 | -0.054 (0.86) | 2213 | -0.18 (0.94) | 1086 | -0.17 (1.16) | 1156 |
| Prudent dietary pattern score | 0.51 (1.065) | 1699 | -0.40 (0.86) | 1838 | -0.32 (0.70) | 2213 | 0.15 (0.93) | 1086 | 0.048 (0.84) | 1156 |

All values are means (standard deviation) unless otherwise specified. s/day: servings per day. 3820 cases (47.1 %) and 4294 controls (52.9 %) were analyzed in the present study. Only individual food items investigated in this study are shown.
