# Supplementary material for: The Effect of Chromosome 9p21 Variants on Cardiovascular Disease May Be Modified by Dietary Intake: Evidence from a Case/Control and a Prospective Study
Source: PLoS Med. 2011 Oct 11;8(10):e1001106. doi: 10.1371/journal.pmed.1001106 (PMC3191151; doi:10.1371/journal.pmed.1001106)
Supplement: Table S2 — Characteristics of the FINRISK participants. All values are proportions unless otherwise specified. (DOC) [file pmed.1001106.s003.doc]

Table S2. Demographic, physical and dietary characteristics of the FINRISK study

|  | **N** | **%** | **Total n** |
| --- | --- | --- | --- |
| CVD incidence | 1014 | 5.3 | 19129 |
| Sex (female) | 10450 | 54.6 | 19129 |
| Age (mean, years) |  | 46.7 (12.6) | 19129 |
| High physical activity | 4179 | 22.1 | 18925 |
| Vegetable consumption, daily | 4971 | 26.1 | 19065 |
| Fruit consumption, daily | 6014 | 31.6 | 19046 |
| Berry consumption, daily | 1366 | 7.2 | 19055 |
| Diet of vegetables, fruits and berries |  |  |  |
| low group | 12314 | 64.4 | 19129 |
| medium group | 3526 | 18.4 | 19129 |
| high group | 3289 | 17.2 | 19129 |

All values are proportions unless otherwise specified.
