# Supplementary material for: The Effect of Chromosome 9p21 Variants on Cardiovascular Disease May Be Modified by Dietary Intake: Evidence from a Case/Control and a Prospective Study
Source: PLoS Med. 2011 Oct 11;8(10):e1001106. doi: 10.1371/journal.pmed.1001106 (PMC3191151; doi:10.1371/journal.pmed.1001106)
Supplement: Text S1 — INTERHEART investigators. (DOC) [file pmed.1001106.s004.doc]

# INTERHEART national coordinators* and investigators

Argentina: R Diaz*, E Paolasso*, M Ciruzzi*, Instituto Cardiovascular de Rosario

R Nordaby, CH Sosa, HL Luciardi, EM Marzetti, JA Piasentin, M Muchastegui, C Cuneo, G Zapata, JO Bono, EM Marxetti, A Caccavo, RAA Guerrero, R Nordaby, C Dizeo;

Australia and New Zealand**:** D Hunt*, J Varigos*, University of Melbourne, Royal Melbourne Hospital

M Sallaberger, G Aroney, P Hicks, D Campbell, J Amerena, W Walsh, G Nelson, D Careless, J Durech, K Gunawardane, A Thomson, D Owensby, A Buncle, B Watson, H Ikram, M Audeau, M Hills, D Rosen, J Rankin, A Tunesi, J Sampson, K Roberts, A Hamer, L Roberts, B Singh, C Singh, R Hendriks, G Tulloch, A Hill, D Rees, C Hall;

Bahrain**:** MA Halim*, Al Khalifa, Cardiac Centre

MI Amin;

Bangladesh**:** S Haque*, Sheik Mujih, Medical University/Institute of Post Grad Medicine

GM Faruque, M Hossain;

Benin Republic**:** H Agboton*, General Teaching Hospital/Medical School;

Botswana**:** C Onen*, Center for Chronic Diseases, Gaborone Private Hospital, Gaborone;

Brazil**:** A Avezum*, L Piegas*, Instituto Dante Pazzanese de Cardiologia, Sao Paulo

G Reis, JP Esteves, LC Bodanese, AR Junior, JAM Neto, RF Ramos, ES Silva, A Labrunie, ACC Carvalho, IP Filho, DCD Albuquerque, JAM Abrantes, OCE Gebara, PE Leaes, SS Xavier, RL Marino, O Dutra;

Cameroon: W Muna*, K N Blackett*, CHU, Yaoundé;

Canada**:** K Teo*, S Yusuf*, Hamilton General Hospital, Hamilton

S Ounpuu, YK Chan, C Joyner, AD Kitching, A Morris, A Panju, H Lee, I Bata, B May, JG Hiscock, PV Greenwood, W Tymchak, M Natarajan;

Chile**:** F Lanas*, Facultad de Medicina, Universidad de la Frontera, Temuco

D Standen, A Lanas, C Santibanez, P Soto, S Potthoff, R Soto, E Mercadal;

China**:** L S Liu*, L X Jiang*, Beijing Hypertension League Institute and Fu Wai Hospital, Beijing

Q Chen, N Sun, J Wang, L Zhang, X Li, H Chen, Z Li, Y Shi, J Meng, D Wang, Z Liu, J Yan, W Zhang, Z Wan, Y Xu, X Li, L Wang, Q Fu, K Zhu, Y Wang, X Wang, F Wang, R Liu, R Zhao, K Sun, S Yue, Y Zhang, X Zhao, L Zhao, C Zhou, X Meng, A Liu, X Li, R Wu, R Xiao, X Zhang, G Wan, X Chen, Z Ge, T Zhou, Y Li, X Zhou, F Zhang;

Colombia**:** LE Bautista*, Fundación Cardiovascular del Oriente Colombiano

JP Casas, CA Morillo, E Ardilla, IA Arenas, M Lindarte, D Aristizabal;

Croatia**:** Z Rumboldt*, University of Split

M Rumboldt, V Carevic;

Czech Republic: P Widimsky*, Charles University, Prague

M Branny, P Gregor;

Egypt**:** MS Khedr*, Suez Canal University, Cario

MG Abdel-Aziz, S Abdel-Kader, MA Abdel-Moneim, F El-Demerdash, A El-Saiid, S Gharib, M Hassanin, F Abdel-Hamid Maklady, I Sadek;

Germany**:** HJ Rupprecht*, T Wittlinger*, Oberarzt der II Medizinische Klinik

P Schuster, A Schmidt, HR Ochs;

Greece**:** N Karatzas*, A Pipilis*, Athens Hygeria SA

D Symeonides, K Karidis, TH Tsaknakis, K Kyfnidis;

Guatemala**:** M Luna*, Unidad de Cirugia Cardiovascular de Guatemala

AM Arroyo, G Sotomora, MA Rodas, L Velasquez, A Ovando;

Hong Kong**:** J Sanderson*, Chinese University of Hong Kong, Prince of Wales Hospital

WY Ma, S Chan;

Hungary**:** M Keltai*, Hungary Institute of Cardiology Budapest

F Szaboki, P Karpati, K Toth, S Timar, E Kalo, A Janosi, M Rusznak, D Lehoczky, J Tarjan, E Kiss, E Sitkei, P Valyi, I Edes;

India**:** P Pais*, KS Reddy*, St. John’s Medical College and All India Institute of Medical Sciences

PP Joshi, D Prabhakaran, D Xavier, A Roy, L Ramakrishnan;

Iran: MRM Hasani*, SH Mirkhani*, Islamic Azad University, Tehran;

Israel: D Halon*, B Lewis*, Lady Davis Carmel Hospital, Haifa;

Italy: MG Franzosi*, G Tognoni*, Department of Cardiovascular Research, Instituto Mario Negri, Milano

E Gardinale, M Villella, M Mennuni, F Saiu, G Pettinati, S Ciricugno, S Antonaci, L Carrieri, E Balli, D Bicego, MG DelleDonne, A Ottaviano, L Moretti, C Melloni, G Melandri, E Carbonieri, A Vetrano;

Japan: M Hori*, Osaka University Graduate School of Medicine, Suita

H Sato, K Fujii;

Kenya: EN Ogola*, Nairobi Hospital

P Wangai;

Kuwait: M Zubaid*, Kuwait University, Kuwait City

W Rashed;

Malaysia: CC Lang*, University Malaya, Faculty of Medicine, Kuala Lumpur

WAW Ahmad, A Kadirvelu, R Zambahari;

Mexico: MA Ramos-Corrales*, Hospital de Especialidades, Mexico City;

Mozambique: A Damasceno*, Maputo General Hospital;

Nepal: M R Pandey*, Nepal Heart Foundation

G Baniya, A Sayami, M Kiratee, B Rawat, M Pandey, J Gurung;

Netherlands: R Peters*, Acadmisch Medisch Centrum

DCG Basart, PNA Bronzwaer, JA Heijmeriks, HR Michels, GMA Pop;

Nigeria: KK Akinroye*, Nigerian Heart Foundation;

Pakistan: K Kazmi*, The Aga Khan University, Karachi

NA Memon, SK Memon, S Nishtar, A Badar, MA Mattu, M Yakub, K Soomra, J Khatri, AMA Faruqui, SI Rasool, A Samad;

Philippines: AL Dans*, Philippines General Hospital

FQ Punzalan, MVC Villarruz, BT Mendoza, CS Recto III, BR Tamesis, V Mendoza, DJ Torres, DD Morales, I Ongtengco;

Poland: A Budaj*, L Ceremuzynski*, Grochowski Hospital, Klinika Kardiologii CMKP, Warsawa

S Stec, J Gorny, K Prochniewska, TK Urbanek, P Wojewoda, M Pawlowski, K Religa, R Klabisz, H Latocha, M Szpajer, K Cymerman, M Laniec, H Danielewicz, M Ogorek, D Kopcik, A Baranowska, A Kozlowski, M Blachowicz, A Jedrzejowski, T Waszyrowski, Z Zielinski, K Janik, M Tomzynski, M Mytnik, A Maziarz, G Rembelska, M Piepiorka, K Debkowska, T Krynski, B S Szczeklik, W Krasowski, M Rozwodowska, P Miekus, J Surwilo, S Malinowski, J Hybel, M Michalak, P Achremczyk, J Majchrzak, W S Korombel, M Dobrowolski, J Gessek;

Portugal: J Morais*, University Hospital, Coronary Care Unit;

Qatar: AA Gehani*, HA Hajar*, Hamad General Hospital

MM Almowla, AA Omer;

Russia: EG Volkova*, Ural State Medical Academy, Chelyabinsk

SU Levashov, OM Filatova, UI Evchenko, GS Malkiman, NN Karaulovskaya;

Seychelles: J Panovsky*, Victoria Hospital;

Singapore: BA Johan*, National Heart Centre

A Cheng, KS Ng, YL Lim, KH Neoh, KS Tan, LN Lum, Y Cheah;

South Africa: P Commerford*, K Steyn*, University of Cape Town

B Brown, F Martiz, P Raubenheimer, A Aboo, JD Marx, E Batiste, K Sliwa, P Sareli, C Zambiakides;

Spain: V Valentin*, Universitari Dr Peset, Valencia

J Ferriz, A Rovira, A Rodriguez-Llorian, E Cereijo, T Monzo, N Alonso, E Gomez-Martinez, A Lopez-Perez;

Sri Lanka: S Mendis*, Peradeniya Teaching Hospital

T Jayalath;

Sultanate of Oman: ATA Hinai*, Sultan Qaboos University, Muscat

MO Hassan;

Sweden: A Rosengren*, Sahlgrenska University Hospital/Ostra, Göteborg;

Thailand: C Sitthi-Amorn*, Department of Medicine, Chulalongkorn University, Bangkok

S Chaithiraphan, K Bhuripanyo, S Srimahachota, M Anukulwutipong, P Loothavorn, P Sritara, S Tanomsup, J Tantitham, P Tatsanavivat, T Yipinsoi, W Jintapakorn, W Puavilai, B Koanantakul, P Kasemsuwan, C Supanantareauk, C Peamsomboon, B Saejueng, K Jeamsomboon, A Sukontasup;

United Arab Emirates: W Almahmeed*, Al-Jazeira and Central Hospitals, Abu Dhabi

LO Abdelwareth, A Bokhari, NS Rao, S Bakir, AM Yusufali, E Hatou, Q Zaidi;

UK: K Fox*, M Flather*, Royal Infirmary of Edinburgh, Edinburgh;

USA: J Probstfield*, University of Washington, Seattle

R Freeman, J Mathew, M Schweiger, W Johnson, E Ofili;

Zimbabwe: J Chifamba*, University of Zimbabwe

AJG Hakim.

*National coordinator.
